# Novel technique of sclerotherapy for superficial lymphatic malformation

**DOI:** 10.3389/fped.2025.1614095

**Published:** 2025-06-27

**Authors:** Hidehito Usui, Masato Shinkai, Satoshi Tanaka, Rento Morishima, Kazuki Shirane, Takafumi Kondo, Kyoko Mochizuki, Norihiko Kitagawa

**Affiliations:** ^1^ Department of Surgery, Kanagawa Children’s Medical Center, Yokohama, Japan; ^2^ Department of Surgery, Yokohama City University, Yokohama, Japan

**Keywords:** superficial lymphatic malformation, lymphatic malformation, sclerotherapy, bleomycin, vascular malformations, pediatric surgery

## Abstract

**Purpose:**

Superficial lymphatic malformation (SLM), previously known as lymphangioma circumscriptum, is characterized by skin lesions that sometimes require interventional treatment. However, an effective treatment has yet to be established. We proposed an effective sclerotherapy procedure based on the pathophysiology of SLM.

**Methods:**

Seven patients with SLM who were treated at our hospital between April 2015 and April 2024 were retrospectively enrolled. To maximize the treatment effects, sclerotherapy with bleomycin targeting the SLM and LM deep beneath the SLM was performed.

**Results:**

Of the seven patients, four were females, and three were males. The SLM sites were mucosal lesions in four cases and skin lesions in three cases. The median age at the first sclerotherapy was 10 (1–18) years. A median of one course (1–3) of sclerotherapy resulted in a marked reduction of SLM lesions on gross evaluation to the satisfaction of the patient. The median observation period was 451 (59–2,901) days. No serious complications occurred, except for transient vomiting in one patient and temporary skin pigmentation in two patients. No patient experienced recurrence requiring retreatment.

**Conclusion:**

Our study suggests that sclerotherapy for the SLM and LM deep beneath the SLM may be an effective treatment for SLM.

## Introduction

Lymphatic malformations (LM) are congenital low-flow vascular anomalies of the lymphatic system that occur in one of 2,000–4,000 live births and typically form mass lesions (1). Seventy-five percent of LM cases occur in the cervicofacial region (2). Superficial lymphatic malformation (SLM), previously known as lymphangioma circumscriptum, is generally considered a congenital LM that can arise in cutaneous or mucosal tissue, mostly located in the head and neck, proximal extremities, trunk, and buttocks (3, 4). However, others have hypothesized that SLM may be a secondary skin lesion induced by the LM deep beneath the SLM rather than a primary LM lesion (5).

SLM is often asymptomatic except for skin lesion eruptions. However, treatment is warranted when complications such as lymphorrhea, bleeding, infection, pain, or cosmetic disturbances arise (6). The reported treatment options for SLM include surgical excision, sclerotherapy, electrocoagulation, liquid nitrogen therapy, and carbon dioxide laser therapy (4). Surgical excision has become a common option for SLM; however, high recurrence rates and complications after surgery are problematic (7, 8). Sclerotherapy is a minimally invasive treatment that has become a well-accepted procedure for LM (9); however, it is not commonly accepted as a treatment for SLM (6). Some studies have demonstrated that sclerotherapy on its own does not yield encouraging results for SLM (10). Therefore, the currently available treatment options for SLM have not yielded favorable outcomes, and methods to improve the quality of life of patients with SLM are warranted.

In this study, we proposed a novel sclerotherapy technique that sequentially targets the deep LM and SLM to maximize the efficacy of sclerotherapy for the SLM.

## Methods

The study included seven patients with SLM who underwent sclerotherapy between April 2015 and April 2024. LM was diagnosed based on clinical manifestations and imaging findings on ultrasonography and magnetic resonance imaging (MRI). This was also pathologically confirmed in two patients. Our sclerotherapy was indicated for patients with symptoms associated with SLM, such as lymphorrhea, bleeding, recurrent swelling, pain, or cosmetic concerns, who opted for interventional treatment. All patients provided informed consent and the study was approved by our Institutional Review Board (IRB No. 1405-08).

### Our novel technique of sclerotherapy procedure

**Figure 1:**
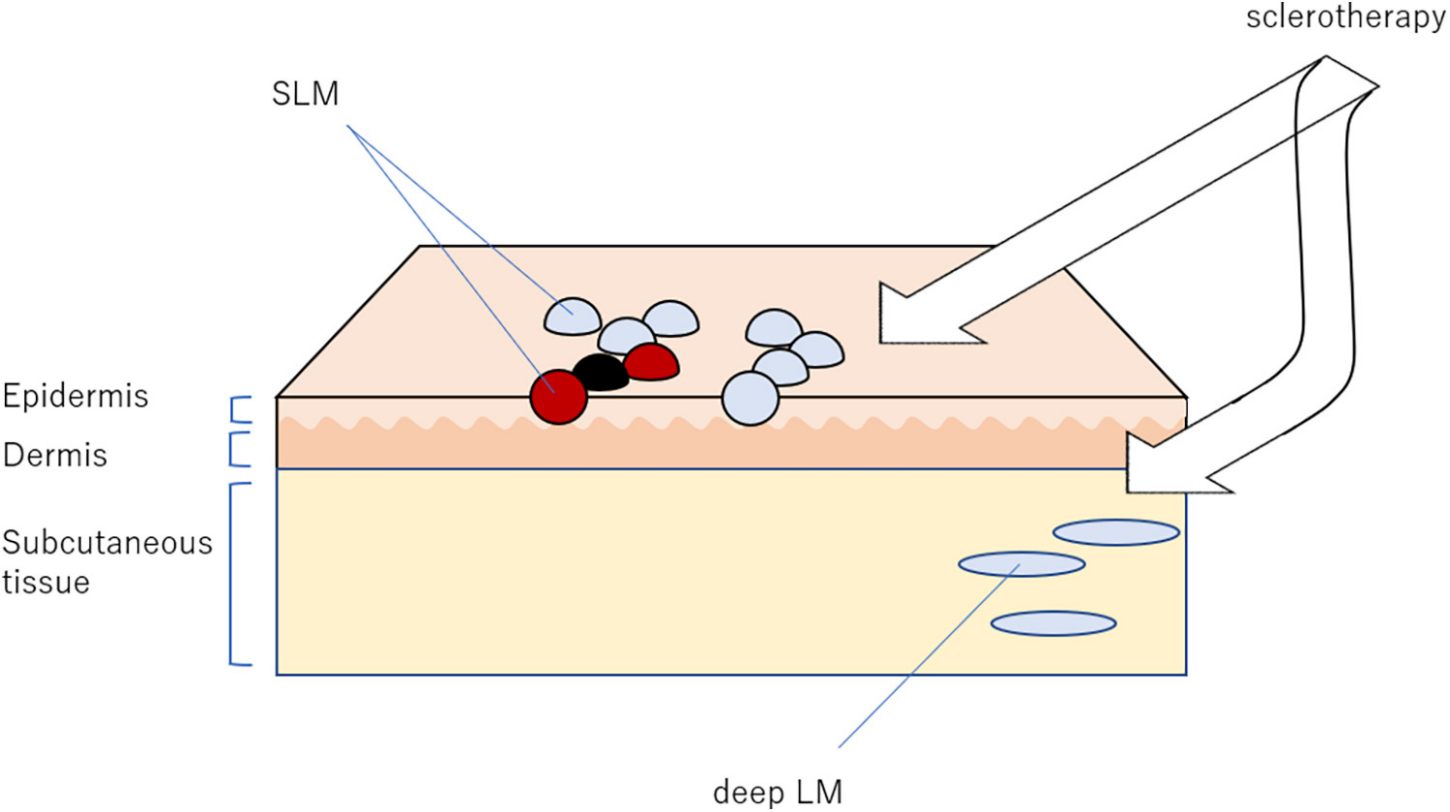
Our sclerotherapy targeting both superficial lymphatic malformation (SLM) and lymphatic malformations (LM). The most important aspect of this treatment is the detection of deep LM, which may present at a short distance from the SLM.

The procedure is illustrated in Figure 1. Prior to treatment, SLM lesions as well as LM lesions in contiguous deeper cutaneous and subcutaneous tissues were identified and their local distribution was visualized using ultrasonography. In cases with mucosal lesions, direct visualization of target lesions from the mucosal surface using ultrasonography can be challenging. However, deep lymphatic malformations associated with mucosal SLM are often accessible transcutaneously using conventional ultrasound. In addition, a small high-frequency ultrasound probe that can be inserted into the oral cavity has recently been introduced, allowing direct application to the mucosal surface and enabling more precise localization of the lesions.

Sclerotherapy was performed under general anesthesia in all patients. First, sclerotherapy was performed for the deeper LM lesions. These cystic components were percutaneously punctured using a 21- or 23-gauge needle under ultrasonographic guidance and injected with bleomycin. When targeting deep LM lesions, all identifiable cysts were punctured and injected whenever possible. If diffusion of bleomycin into adjacent cysts was observed during injection, suggesting interconnection, additional puncture of those cysts was omitted. Subsequently, sclerotherapy was performed for the SLM lesions. For SLM lesions, direct puncture from the surface was avoided to minimize the risk of rupture and bleomycin leakage. Instead, a 26-gauge needle was inserted from a skin site slightly distant from the lesion and advanced through the subcutaneous tissue to reach the base of the target cyst from beneath. Ultrasound guidance was not used during this particular step. This technique prevents cyst rupture and bleomycin leakage from the SLM cysts. The vesicles visibly expanded or became distended upon proper injection, confirming successful delivery of bleomycin.

We selected bleomycin as the sclerosing agent because it has shown promising efficacy for microcystic lymphatic malformations, which include most cases of SLM (11–14). Bleomycin (Bleo®, Nippon Kayaku Co., Ltd., Tokyo, Japan) was obtained as a lyophilized powder and reconstituted with normal saline to a concentration of 1 mg/ml prior to administration. A total dose ranging from 0.25 to 0.6 mg/kg with a maximum of 5 mg of bleomycin was injected, based on our institutional protocol, which was developed and previously reported in accordance with studies on LMs (9).

After the procedure, all patients were observed under inpatient care for approximately 48 h to monitor for potential adverse events. Adverse events were assessed according to the Common Terminology Criteria for Adverse Events (CTCAE) version 5.0. Severe complications were defined as adverse events of Grade 3 or higher.

Sclerotherapy was performed at intervals of at least several months, allowing time to evaluate the therapeutic effect of the drug. The decision to repeat or terminate treatment was based on the persistence of symptoms, the patient's or guardian's preference, and clinical findings regarding lesion size or appearance.

Treatment efficacy was evaluated based on clinical findings and patient-reported outcomes, including resolution of symptoms, reduction in lesion size or appearance, and satisfaction reported by the patient or guardian.

## Results

Table 1 shows patient characteristics. Four patients were females and three were males. The SLM sites were mucosal lesions in four patients (two on the tongue and two in the oral cavity) and skin lesions in three patients (one on the neck and two on the lumbar back). Three patients had a history of sclerotherapy in infancy for LM without SLM, and one had undergone two recent surgeries for SLM. The median age at the first sclerotherapy was 10 years (range, 1–18 years). A median of one course of sclerotherapy (range, 1–3 courses) resulted in a marked reduction of SLM lesions on gross evaluation to the satisfaction of the patient. The median observation period was 451 days (range, 59–2,901 days). No serious complications occurred, except for transient vomiting in one patient and temporary skin pigmentation in two patients. No recurrence requiring retreatment was observed.

**Table 1:** Patient characteristics.

Case	Sex	Age	Site of LC	Past treatment of LM not LC (times)	Past treatment of LC (times)	Courses of sclerotherapy discussed here	Observation period (days)
1	F	1	mucosa (tongue)	–	–	1	2,901
2	F	18	skin (lumbar back)	OK432 (1)	–	2	1,021
3	F	13	skin (cervix)	OK432 (3)	surgical excision (2)	3	832
4	M	10	mucosa (tongue)	–	–	1	451
5	M	11	skin (lumbar back)	–	–	1	306
6	M	6	mucosa (lip, oral cavity)	–	–	2	222
7	F	4	mucosa (oral cavity)	bleomycin (3)	–	1	59
median		10				1	451

The detailed clinical courses of three representative cases are described below.

### Case 3

**Figure 2:**
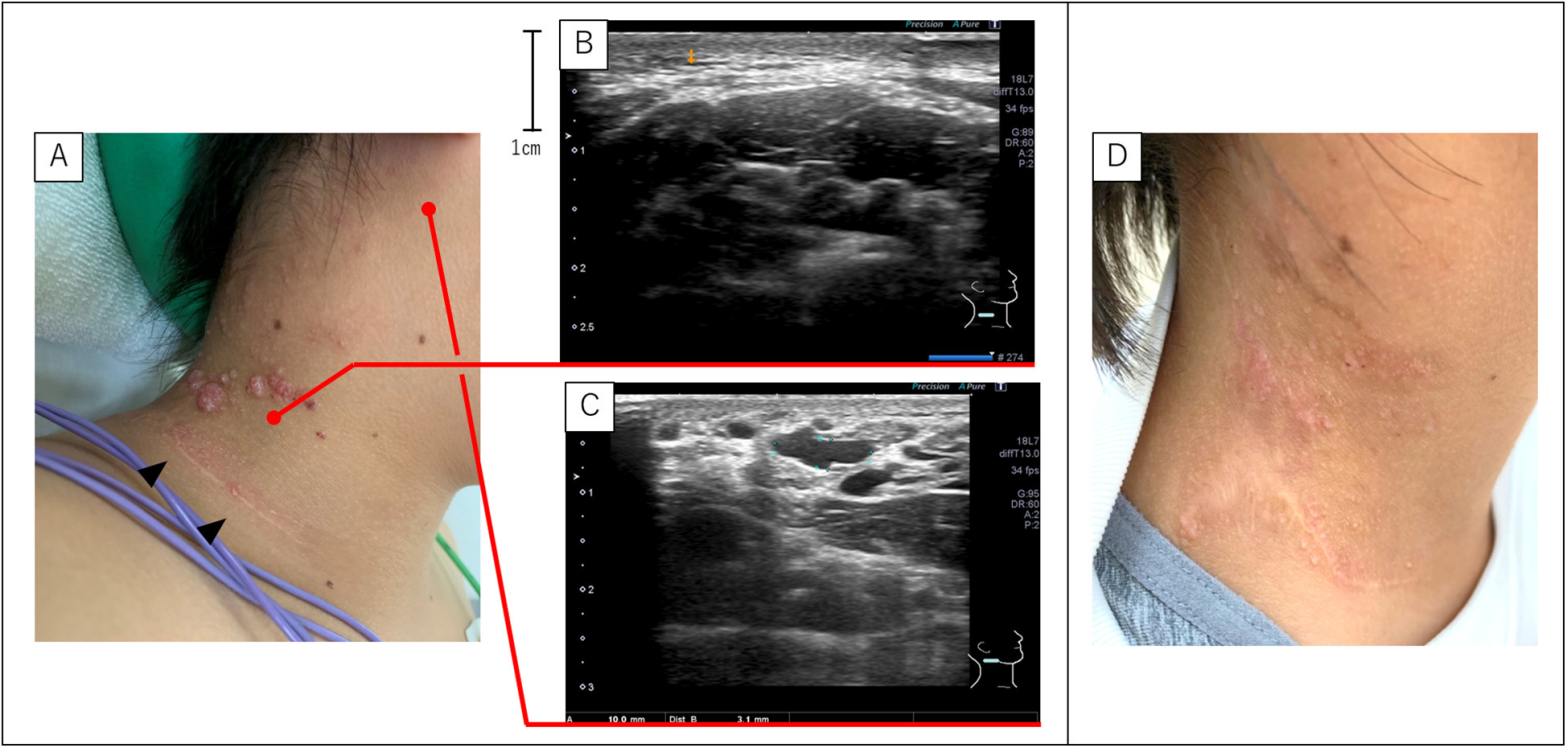
SLM and LM of case 3. **(A)** Gross appearance of the SLM before sclerotherapy. The SLM appeared on the cephalic side of the surgical scar (black arrowheads) at the previous hospital. **(B,C)** Ultrasonography detected no LM directly under the SLM. However, the LM was present near the lower edge of the ear and distant from the SLM. **(D)** Post-sclerotherapy findings show that the SLM is almost unidentifiable. LM, lymphatic malformations; SLM, superficial lymphatic malformation.

A 13-year-old girl with cervical SLM was referred to our hospital. At a previous hospital, two courses of sclerotherapy with picibanil (OK-432) for LM without SLM were administered during toddlerhood. Later, SLM appeared and skin containing the SLM was surgically excised at 10 years of age, which failed to prevent recurrence. A second SLM excision was performed; however, the lesion recurred again. During her visit to our hospital, a surgical scar was visible on her neck, with multiple SLMs on the cephalic side (Figure 2A). Notably, no LM was found directly beneath the SLM (Figure 2B); however, detailed ultrasonographic evaluation revealed a deep microcystic LM under the posterior occipital skin near the SLM lesion (Figure 2C). Therefore, sclerotherapy targeting both SLM and LM was performed. After two courses of sclerotherapy, the patient's symptoms markedly improved (Figure 2D). She then requested a third course with the expectation of further improvement. After the second course, the patient vomited several times but recovered within a few days.

### Case 4

**Figure 3:**
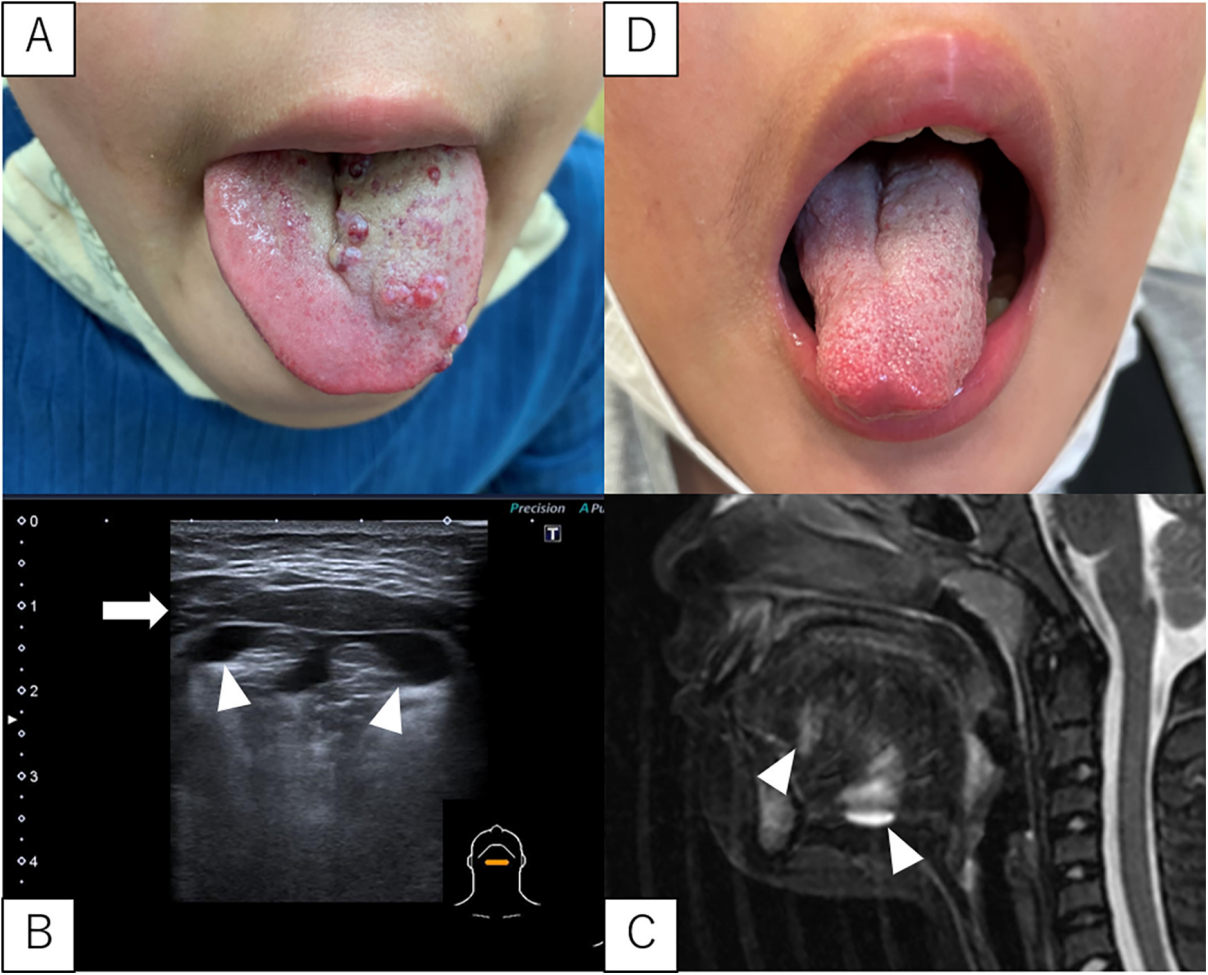
SLM and LM in case 4. **(A)** Pre-sclerotherapy gross appearance of the SLM on the tongue. **(B,C)** Ultrasonography (from below the mandible) and MRI (T2, sagittal section) showing a deep LM in the mylohyoid muscle and tongue. White arrowheads indicate the LM. The white arrows indicate the mylohyoid muscle. **(D)** Gross appearance after sclerotherapy SLM are almost unidentifiable. LM, lymphatic malformations; SLM, superficial lymphatic malformation; MRI, magnetic resonance imaging.

A 10-year-old boy was referred to our hospital because of tongue SLM (Figure 3A). Since birth, his tongue occasionally became swollen and SLM became more prominent at the age of six. The ultrasonography from below the mandible revealed an LM deep in the mylohyoid muscle as well as in the tongue (Figure 3B). MRI confirmed these findings (Figure 3C). Sclerotherapy targeting both the SLM and deep LM was performed, and the symptoms resolved after one course (Figure 3D).

### Case 6

**Figure 4:**
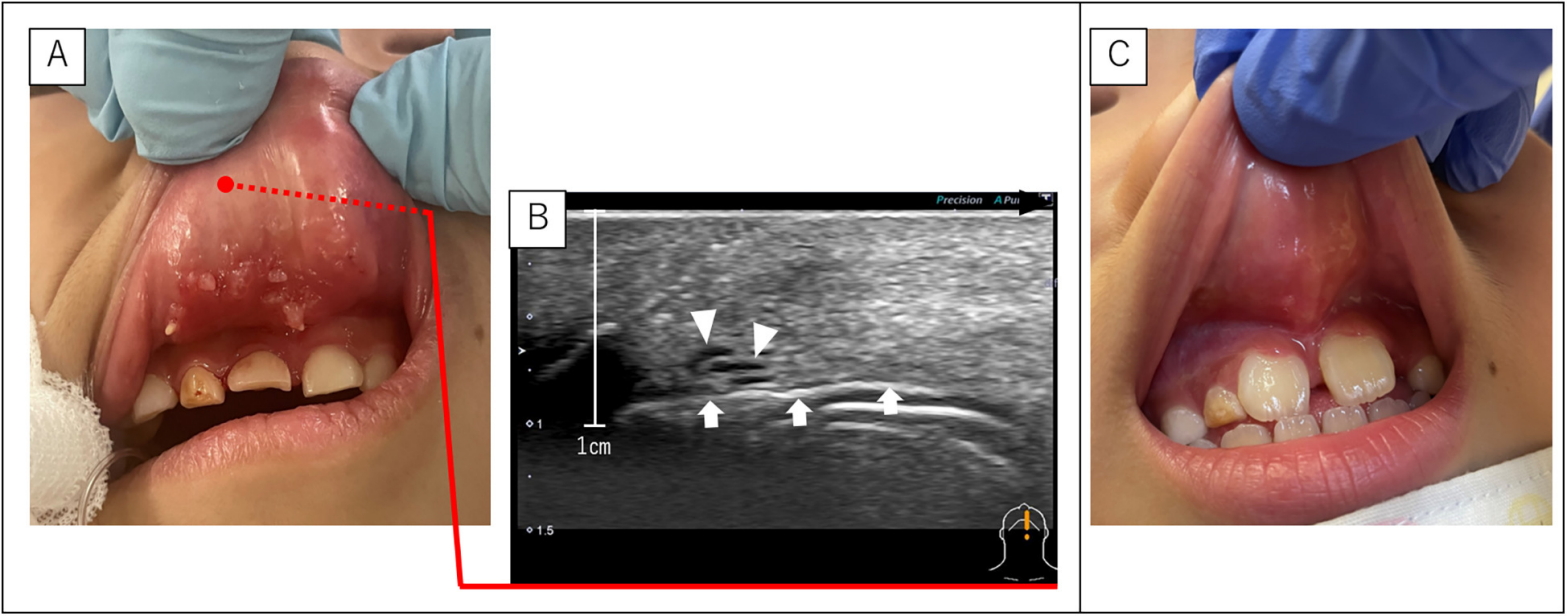
SLM and LM of case 6. **(A)** Pre-sclerotherapy findings showing SLM on the back of the upper lip. **(B)** Ultrasonography (sagittal section) showing a fine LM on the upper lip closest to the nose. The black arrowhead indicates the direction of the lip edge (caudal side). White arrowheads indicate the LM. White arrow indicates air in the oral cavity. **(C)** Post-sclerotherapy findings show that the SLM is almost unidentifiable. LM, lymphatic malformations; SLM, superficial lymphatic malformation.

A 6-year-old boy was referred for upper lip swelling and SLM of the inner lip (Figure 4A). A biopsy performed at a previous hospital diagnosed the lesion as an LM. In addition, ultrasonography revealed a microcystic LM deep within the upper lip closest to the nose (Figure 4B). Sclerotherapy targeting both SLM and LM was performed, and the symptoms resolved after one course (Figure 4C).

## Discussion

LMs are defined as malformations of the lymphatic vessels consisting of thin-walled, irregularly shaped lymphatic spaces of varying sizes lined with lymphatic endothelial cells (15). The International Society for the Study of Vascular Anomalies (ISSVA) classifies LMs into three types: macrocystic, microcystic, and mixed cystic (16). SLMs are considered cutaneous LMs that can appear on any cutaneous surface or mucous membrane (3). Histologically, the SLMs are similar to the LM. Clinically, a common characteristic of SLM is the presence of a group of transparent vesicles 2–4 mm in size with patterns resembling frog spawn, which are diverse in color owing to hemoglobin degradation (3, 17). Due to its small vesicles, SLM is classified as microcystic LM according to the ISSVA classification (10). Although dermatoscopy and biopsy with immunostaining have reportedly been useful for the diagnosis (3, 17), most SLMs can be diagnosed based on clinical manifestations alone.

Unlike the high risk of cervical LM around the airway (9), SLM is not life threatening by itself; however, it can cause chronic complications in some patients (6). Spontaneous resolution of SLM is rare; therefore, surgical excision is often indicated (7, 8) However, surgery carries the risk of complications including infection, nerve injury, cosmetic deformity, and a relatively high recurrence rate of up to 25% (7). Therefore, a less-invasive treatment approach is required. Sclerotherapy is usually the first treatment choice for cervical LM because it is inexpensive, minimally invasive, repeatable, associated with fewer complications, and comparable efficacy to surgery (18, 19). However, there have been only a few studies on the efficacy of sclerotherapy in SLM, and some reports have shown that it does not yield encouraging results alone (6, 7, 10).

The most important aspect of this treatment is the detection of deep LM using ultrasonography. In most cases of SLM, deep subcutaneous LM lesions are present simultaneously and can be detected through a careful ultrasonography examination. They are occasionally located directly under the SLM lesion and more frequently at short distances from the SLM lesion. Our novel sclerotherapy technique effectively targets deep LM and SLM lesions. Our treatment strategy is consistent with the resection strategy; complete removal of the deep lesion is essential because resection of only the superficial lesion often results in recurrence (6, 8), as shown in our study, which included one patient with SLM recurrence shortly after resection at a previous hospital. Interestingly, the recurrence site was observed to be located upstream of the lymphatic flow from the scar, suggesting that SLM recurrence may be caused by surgical disruption of the lymphatic flow from a deep LM lesion, followed by the eruption of the lymphatic fluid into the skin upstream from the scar. These findings shed light on a possible pathogenesis of SLM, which is distinct from that of LM. In 1976, Whimster originally hypothesized that SLM might be a secondary lesion arising from a deep LM (5, 8). In addition, a previous report on the difference in gene expression between SLM and LM suggested that SLM may occur secondary to LM rather than being a true LM, which further supports the above hypothesis (20). Determination of whether SLM harbors phosphatidylinositol-4,5-bisphosphate 3-kinase catalytic subunit alpha (*PIK3CA)* mutations or not may help identify the pathogenic differences between SLM and LM, as *PIK3CA* mutations that are pathognomonic of LM have garnered the attention of researchers in recent years (21, 22). From a therapeutic perspective, there are many reports of high SLM recurrence after inadequate intervention of deep LM, and treating deep LM lesions may be vital in preventing SLM recurrence. Khurana et al. reported a combination of sclerotherapy and radiofrequency ablation to target superficial and deeper lesions, respectively, which is the same strategy used in our study (6). Wang et al. reported a combination of liposuction and sclerotherapy for deep LM (23). However, ultrasonography-guided sclerotherapy has become well accepted as a standard treatment for cervical LM; sclerotherapy for both deep and superficial lesions is a simple and inexpensive extension of this procedure, although careful ultrasonographic evaluation is required to detect deep lesions.

Sclerotherapy agents used for LMs in previous studies included picibanil, doxycycline, bleomycin, ethanol, sodium tetradecyl sulfate, and hypertonic saline. At present, there is no consensus regarding the most effective sclerotherapy agent (24, 25). While microcystic LMs have generally been more difficult to treat than macrocystic ones, bleomycin has shown promising efficacy in several studies (11–14). Most SLMs are classified as microcystic LMs; therefore, bleomycin was used in our study. The mechanism of action of bleomycin in LMs is not yet fully elucidated; however, it is believed to involve endothelial damage and subsequent fibrosis, in addition to its known cytotoxic effects (13).

An adverse effect of this therapy was local skin pigmentation in two patients, which persisted for approximately 1 month but disappeared after a few months. We should be vigilant about pulmonary fibrosis, a serious dose-dependent complication of bleomycin, although this complication is very rare, and we encountered no such case in our study (26).

This study has several limitations. First, the retrospective nature of the case series may introduce selection bias. In addition, although our technique was effective for small to moderately sized SLM, it may be less effective for extensive lesions or those with fibrotic consolidation. In such cases, curettage combined with sclerotherapy may be a more appropriate option (27).

## Conclusion

Our novel sclerotherapy technique targeting both the SLM and deep LM is an effective treatment for SLM.

## Data Availability

The original contributions presented in the study are included in the article/Supplementary Material, further inquiries can be directed to the corresponding author.
